# Antitumor Activity of s-Triazine Derivatives: A Systematic Review

**DOI:** 10.3390/molecules28114278

**Published:** 2023-05-23

**Authors:** Qiuzi Dai, Qinsheng Sun, Xiaorong Ouyang, Jinyang Liu, Liye Jin, Ahao Liu, Binsheng He, Tingting Fan, Yuyang Jiang

**Affiliations:** 1 The Hunan Provincial University Key Laboratory of the Fundamental and Clinical Research on Functional Nucleic Acid, Hunan Key Laboratory of the Research and Development of Novel Pharmaceutical Preparations, Changsha Medical University, Changsha 410219, China; dqz19@tsinghua.org.cn (Q.D.); xiaorongouyang123@163.com (X.O.); liujinyang003326@163.com (J.L.); jinliye06@gmail.com (L.J.); haoxc1215@163.com (A.L.); 2 State Key Laboratory of Chemical Oncogenomics, Tsinghua Shenzhen International Graduate School, Shenzhen 518055, China; sunqinsheng@163.com; 3 Institute of Biomedical Health Technology and Engineering, Shenzhen Bay Laboratory, Shenzhen 518132, China; 4 School of Pharmaceutical Sciences, Tsinghua University, Beijing 100084, China

**Keywords:** s-triazines, antitumor, drug target

## Abstract

1,3,5-triazine derivatives, also called s-triazines, are a series of containing-nitrogen heterocyclic compounds that play an important role in anticancer drug design and development. To date, three s-triazine derivatives, including altretamine, gedatolisib, and enasidenib, have already been approved for refractory ovarian cancer, metastatic breast cancer, and leukemia therapy, respectively, demonstrating that the s-triazine core is a useful scaffold for the discovery of novel anticancer drugs. In this review, we mainly focus on s-triazines targeting topoisomerases, tyrosine kinases, phosphoinositide 3-kinases, NADP+-dependent isocitrate dehydrogenases, and cyclin-dependent kinases in diverse signaling pathways, which have been extensively studied. The medicinal chemistry of s-triazine derivatives as anticancer agents was summarized, including discovery, structure optimization, and biological applications. This review will provide a reference to inspire new and original discoveries.

## 1. Introduction

**Figure 1:**
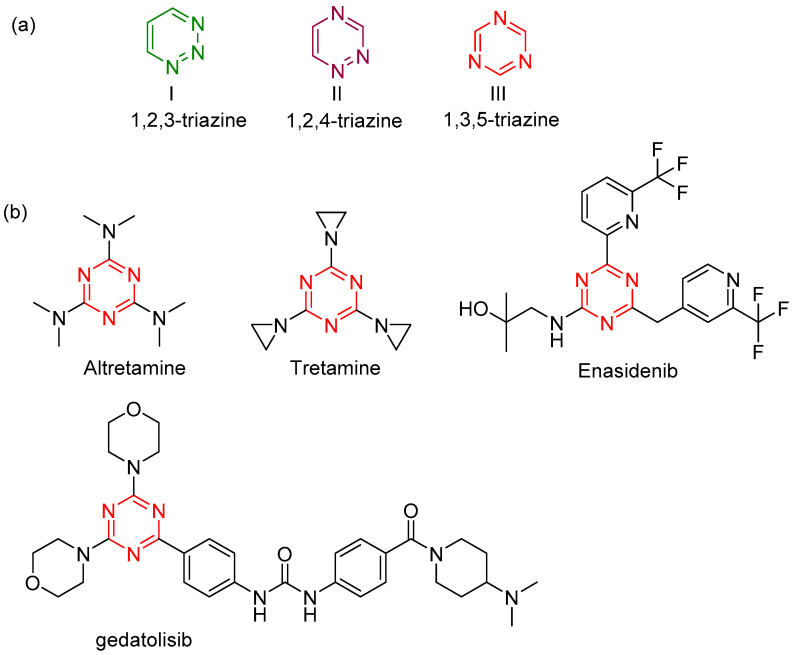
(**a**) Triazine systems; (**b**) some representative commercial drugs of the s-triazine.

Triazines are an important class of heterocyclic compounds. The triazine structure is a heterocyclic, six-membered benzene ring with three carbons replaced by nitrogens. The isomers of triazine (Figure 1a) are distinguished from each other by the positions of their nitrogen atoms and are called 1,2,3-triazine (I), 1,2,4-triazine (II), and 1,3,5-triazine. 1,3,5-triazine, also called s-triazine, has three easily modified sites at positions 2, 4, and 6, usually used for modulating its physicochemical and biological activities. s-Triazine scaffold provides the source for the design of biologically relevant molecules with well-known applications, such as antibacterial, anticancer, and anti-inflammatory [1,2]. s-triazine derivatives have attracted attention due to their remarkable activity against a wide range of cancer cells. As shown in Figure 1b, hexalen [3], also called altretamine, is an example of an antineoplastic drug based on an s-triazine-privileged structure that was first approved by the FDA in 1990 for the treatment of refractory ovarian cancer. After that, various anticancer drugs containing the s-triazine motif were approved, such as tretamine, gedatolisib, and enasidenib. Current studies investigating s-triazine candidates with great promise for antitumor activity focus on different protein kinases, such as topoisomerases, tyrosine kinases, phosphoinositide 3-kinases (PI3K), NADP^+^-dependent isocitrate dehydrogenases (IDH), and cyclin-dependent kinases (CDK).

This study outlined recent progress in s-triazines’ chemical properties, with a focus on the inhibition of the above-mentioned protein kinases. The structure-activity relationships (SARs) of s-triazines elucidated that s-triazines were useful templates for the development of novel drugs for cancer therapy. According to the structure of the active region of the protein kinases, di-substituted s-triazine derivatives mainly inhibit topoisomerase, Bruton’s tyrosine kinase, focal adhesion kinase, and cyclin-dependent kinases, while tri-substituted s-triazine derivatives mainly inhibit epidermal growth factor receptor, phosphatidylinositol-3-kinase, NADP^+^-dependent isocitrate dehydrogenases, and heat shock protein 90. Additionally, the six-membered nitrogen-containing basic heterocycles such as morpholine, piperidine, and piperazine on the triazine moiety were beneficial for its antitumor activity.

This review aims to summarize the recent developments in the innovation of 1,3,5-triazine derivatives as potent antitumor agents. The SAR of these derivatives will be discussed to pave the way for the development of new, safe, and economical antitumors in the near future.

## 2. The Antitumor Activity of s-Triazine Derivatives

### 2.1. Topoisomerases Inhibition

In recent years, a number of DNA topoisomerase inhibitors have been reported in both basic research and clinical applications. Topoisomerase was first discovered in *E. coli* by Wang et al. in 1971. Since then, the study of topoisomerase’s structure, function, and mechanism has been initiated. Generally, human DNA topoisomerases are classified into two large groups: type I and type II topoisomerases. Type I topoisomerases could generate transient single-strand breaks (SSBs) in the DNA molecule by forming a covalent phosphotyrosyl linkage without ATP. In contrast, type II topoisomerases are large homodimeric proteins that require ATP for overall catalytic activity, which could generate transient DNA double-strand breaks (DSBs) [4]. Thus, topoisomerases play important roles in several cell processes, such as replication, transcription, chromosome separation, and segregation. Therefore, topoisomerases are ideal targets for the development of antitumor drugs.

Human DNA topoisomerase inhibitors could be classified into two categories based on their inhibition modes: topoisomerase poisons and topoisomerase catalytic inhibitors [5]. Topoisomerase poisons cover the majority of clinical antitumor agents (e.g., etoposide, doxorubicin, mitoxantrone, salvicine, and teniposide), which can kill cancer cells by stabilizing the covalent topo-DNA complexes and transforming this enzyme into a cellular toxin. Topoisomerase catalytic inhibitors can kill tumor cells by inhibiting the essential enzymatic activity of topoisomerase. A number of structurally diverse compounds have been identified as catalytic inhibitors: preventing DNA cleavage (e.g., merbarone), blocking the enzyme on the ATP-binding site (e.g., purine analogues), or inhibiting ATP hydrolysis (e.g., bisdioxipiperazine analogues). Because of the ubiquitous role of topoisomerase in correlation with various carcinomas, intensive efforts attempting to explore effective tumor therapeutics have led to a panel of chemical agents with diversified structures. In this review, we focused on s-triazine derivatives as topoisomerase inhibitors.

**Figure 2:**
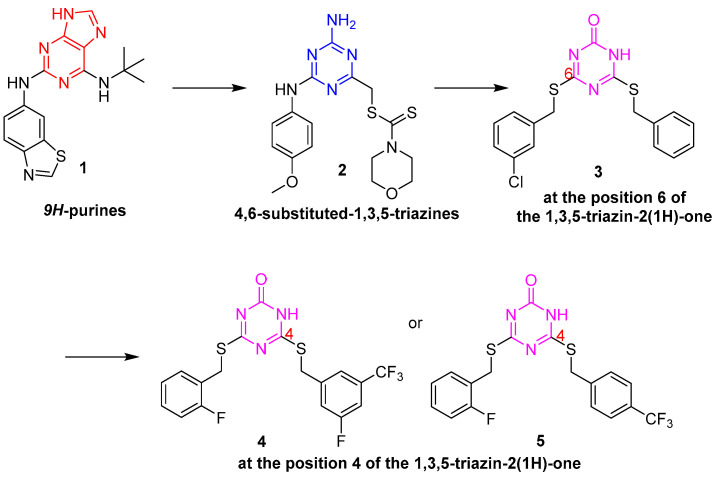
Chemical structures of topoisomerase II inhibitors.

The Novartis research group reported a kind of ATP-competitive catalytic topoisomerase II inhibitor **1** with the core 9H-purine scaffold in 2009 [6], as shown in Figure 2. Then the 9H-purine motif of the compounds was monocyclically substituted by 1,3,5-triazines, which was first reported [7] by Perdih et al. using a virtual screening protocol. In the hit selection, 4-amino-6-(phenylamino)-1,3,5-triazines **2** displayed potent topoisomerase IIa inhibitory activity with IC_50_ values in the micromolar range (IC_50_ = 229 µM). Moreover, compound **2** exhibited cytotoxicity against the HepG2 cell lines (IC_50_ = 20.53 µM), MCF-7 (IC_50_ = 129.0 µM), and normal HUVEC cells (IC_50_ = 122 µM). Furthermore, Perdih et al. designed a series of 4,6-disubstituted-1,3,5-triazin-2(1H)-one analogs [8]. This research provided valuable structure-activity relationship (SAR) data regarding the role of substituents introduced at position 6 of the 1,3,5-triazin-2(1H)-one core. Among which, 6-(benzylthio)-4-((3-chlorobenzyl)thio)-1,3,5-triazin-2(1H)-one **3** was identified as a topoisomerase II inhibitor with an improved IC_50_ of 57.6 µM. However, these compounds showed low cytotoxicity in cancer cells. Perdih et al. further optimize the substituents at position 4 of the 1,3,5-triazin-2(1H)-one scaffold to improve the inhibition potency that would display activity on the cellular level. After virtual screening and experimental evaluation for human topo IIa inhibition, the most potent compounds were **4** and **5,** with IC_50_ values of 8.1 µM and 11.1 µM, respectively, as well as being superior to the positive drug etoposide (IC_50_ = 28.6 µM) [9]. Additionally, compound **5** showed better HepG2 cell inhibitory activity with EC_50_ values of 38.7 µM.

### 2.2. Tyrosine Kinases Inhibition

Tyrosine kinases are implicated in tumorigenesis and progression and have emerged as major targets for drug discovery. Tyrosine kinase inhibitors (TKIs) inhibit corresponding kinases from phosphorylating tyrosine residues of their substrates and then block the activation of downstream signaling pathways. Over the past several decades, multiple robust and well-tolerated TKIs with single or multiple targets, including BTK, EGFR, FAK, VEGFR, ALK, ROS1, HER2, MET, MEK, FGFR, and PDGFR, have been developed. In this section, we summarize the progress of 1,3,5-triazine compounds as TKIs for cancer therapy.

#### 2.2.1. BTK-TKIs

Bruton’s tyrosine kinase (BTK) is the first Tec family tyrosine kinase and also a key member of the B-cell receptor signalling pathway [10]. Since BTK was confirmed to play a crucial role in B-cell maturation as well as in mast cell activation through the high-affinity IgE receptor, studies of BTK as a target have attracted substantial attention from drug researchers, resulting in the development of a diverse array of BTK inhibitors. BTK inhibitors can be divided into reversible and irreversible inhibitors. Most published BTK inhibitors are irreversible inhibitors, including imidazopyrimidine, 2,4-diaminopyrimidines, imidazoquinoxaline, 1,3,5-trazine, and other groups. There are five covalent irreversible BTK inhibitors (Ibrutinib, Acalabrutinib, Zanubrutinib, Tirabrutinib, Orelabrutinib) that have been marketed so far. Irreversible BTK inhibitors usually react with the key amino acid residue Cys481 of BTK via covalent bond modification. However, they may also inhibit other kinases with structurally related cysteines (such as EGFR, BMX, ITK, and JAK3), resulting in off-target effects and potential toxicity after prolonged administration. Meanwhile, several potent noncovalent reversible inhibitors have also been reported, including ARQ-531, Fenebrutinib, Vecabrutinib, and BIIB068, but none of these reversible BTK inhibitors has yet been approved to date.

**Figure 3:**
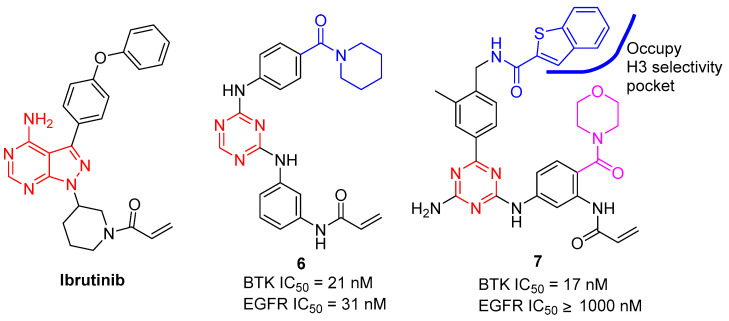
Chemical structures of BTK inhibitors.

Xiang et al. designed a kind of novel covalent BTK inhibitor by substituting the 1H-pyrazolo[3,4-d] pyrimidine core (Ibrutinib) with a 1,3,5-trazines scaffold containing a key acrylamide warhead, as shown in Figure 3. As reported, compound **6** showed potent BTK inhibitory activity (BTK IC_50_ = 21 nM), validating the potential of the 1,3,5-trazine scaffold as a new skeleton for developing effective BTK inhibitors [11]. However, **6** also potently inhibited EGFR with an IC_50_ value of 31 nM. In order to improve its selectivity for BTK over EGFR, the 1,3,5-triazine scaffold modified by more hydrophobic side chains, which would additionally occupy the H3 pocket of BTK, was designed. Structure-activity relationship (SAR) and extensive pharmacological screening led to the discovery of a potent irreversible BTK inhibitor **7** [12]. Compound **7** possessed desirable BTK selectivity (IC_50_ = 17.0 nM) over EGFR (IC_50_ > 1000 nM) and JAK3 (IC_50_ = 104.6 nM), which shared a high degree of homology with BTK. However, it is worth noting that broad kinase panel screening is further needed to clarify the selectivity profile of **7**.

#### 2.2.2. EGFR-TKIs

The epidermal growth factor receptor (EGFR) is a trans-membrane protein belonging to the erbB/HER family of tyrosine kinase (TK) receptors. EGFR signalling in tumour cells, as opposed to normal cells, is changed and often becomes dysregulated. Accordingly, interrupting EGFR communicating signals is considered one of the targets for curing tumors. Multiple EGFR suppressors have been developed, including 1,3,5-triazine derivatives.

**Figure 4:**
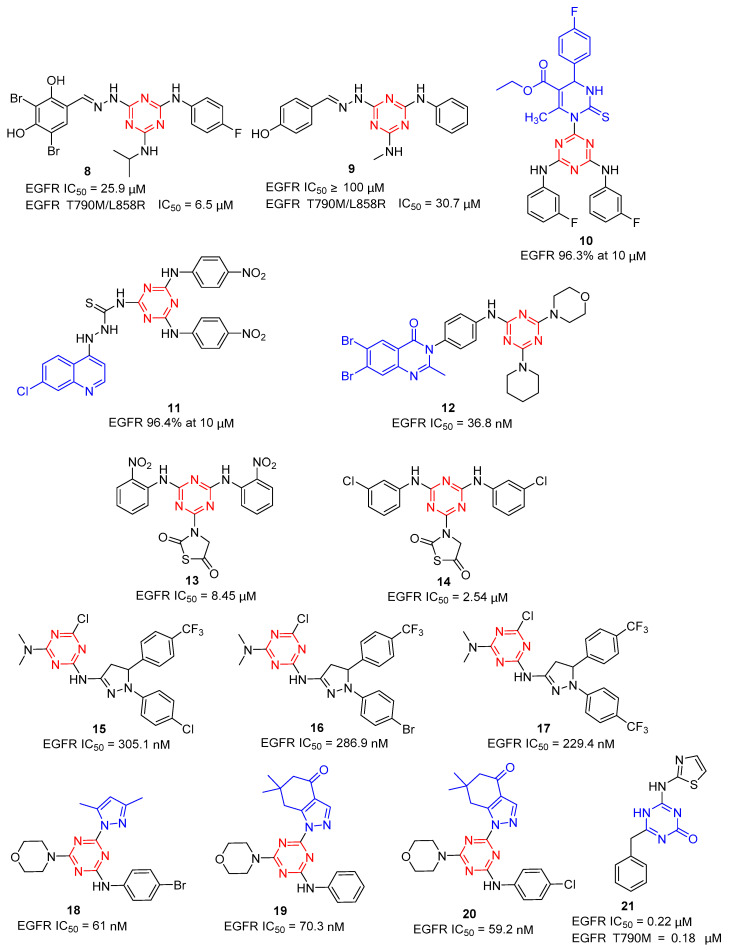
Chemical structures of EGFR inhibitors.

Based on virtual screening, Bai et al. identified a number of 1,3,5-triazine derivatives as potent dual-effective inhibitors against both wild-type and mutant EGFR in 2012 [13]. As shown in Figure 4, compound **8** exhibited the most potent activity against wild-type EGFR (IC_50_ = 25.9 µM) and mutant EGFR T790M/L858R (IC_50_ = 6.5 µM). Structure–activity relationships (SAR) were analyzed among the triazine analogs. Compound **9** was obtained by removing the fluorine group in the phenyl part along with the inclusion of the para-hydroxy group on another phenyl, with a low inhibitory potency for wild-type EGFR (IC_50_ > 100 µM) and reduced about fivefold for mutant EGFR T790M/L858R (IC_50_ = 30.7 µM). Singh groups reported a novel hybrid analogue of monastrol-1,3,5-triazine **10** as an effective anti-breast cancer agent [14] in 2017, which showed excellent EGFR-TK enzyme inhibitory activity (96.3% at 10 µM). Additionally, their group further modified 1,3,5-triazine to obtain 4-aminoquinoline-1,3,5-triazine derivatives [15]. Compound **11** showed the most EGFR-TK enzyme inhibitory activity (96.4% at 10 µM) and excellent anticancer activities against the entire tested cell lines. In 2021, hybrid quinazoline-1,3,5-triazine derivatives as EGFR inhibitors were reported by Pathak [16]. Through pharmacological evaluation, they found that the substitution of the 1,3,5-triazine ring by morpholine and aniline rings could increase the ligand’s potency in EGFR inhibition. Among them, the most active compound **12** showed an IC_50_ value of 36.8 nM against the EGFR enzyme.

In 2018, He et al. investigated four previously reported 1,3,5-triazine compounds as anti-breast cancer agents via modulation of EGFR-TK [17]. Among the compounds, **13** (8.45 ± 0.65 µM) and **14** (2.54 ± 0.22 µM) significantly inhibited EGFR-TK and exhibited potent anticancer activity in all the tested cell lines in a dose-dependent manner. In 2021, 1,3,5-triazine-based pyrazole derivatives were synthesized by Osman et al. with anticancer activity targeting EGFR [18]. Compounds **15**, **16,** and **17** displayed excellent activity with an IC_50_ value of 305.1, 286.9, and 229.4 nM, respectively, in comparison with reference erlotinib.

In 2022, El-Faham et al. synthesized a new series of mono- and bis(dimethylpyrazolyl)-s-triazine derivatives [19]. Of these compounds, **18** showed excellent potency against the HCT116 cell lines (IC_50_ = 500 ± 80 nM) through apoptosis induction and exhibited potent EGFR inhibitory activity with an IC_50_ value of 61 nM (tamoxifen, IC_50_ = 69 nM). For the downstream pathway of PI3K/AKT/mTOR, **18** showed remarkable inhibitory activity with a 0.18-, 0.27-, and 0.39-fold decrease in their concentration. Additionally, pyrazole and fused pyrazole-s-triazine derivatives **19** and **20** were obtained by a one-pot synthesis method, which displayed EGFR inhibitory activity with IC_50_ values of 70.3 and 59.24 nM, respectively, and similar PI3K/AKT/mTOR inhibitory activity compared with **18** [20]. Therefore, compounds **18**, **19,** and **20** possessed EGFR/PI3K/AKT/mTOR signaling cascade inhibitors.

EGFR signaling dysregulation in tumors could result in EGFR overexpression or a gain-of-function mutation. According to the reports, acquired drug resistance to the first-generation EGFR-TKIs was revealed due to the T790M and L858R mutations in EGFR. Although a drug related to EGFR^T790M^ or EGFR^T790M/L858R^ mutation has been discovered, it is still needed to discover new EGFR inhibitors with high selectivity for EGFR^T790M^ and EGFR^T790M/L858R^ kinases. Azmy et al. reported a series of 1,3,5-triazine-based derivatives as potential EGFR^WT^ and EGFR^T790M^ inhibitors [21]. Among the derivatives, compound **21** as the most potent antiproliferative agent showed inhibitory activity with EGFR^WT^ (0.22 ± 0.05 µM) and EGFR^T790M^ (0.18 ± 0.11 µM), compared to the reference drugs erlotinib (0.09 ± 0.05 µM) and AZD9291 (0.55 ± 0.10 µM).

#### 2.2.3. FAK-TKIs

Focal Adhesion Kinase (FAK) is an intracellular non-receptor tyrosine kinase that plays a crucial role in reproduction, early embryonic development, and tumorigenesis through its kinase activity and scaffold function. So far, s-triazines have been proven to be effective inhibitors targeting FAK [22].

**Figure 5:**
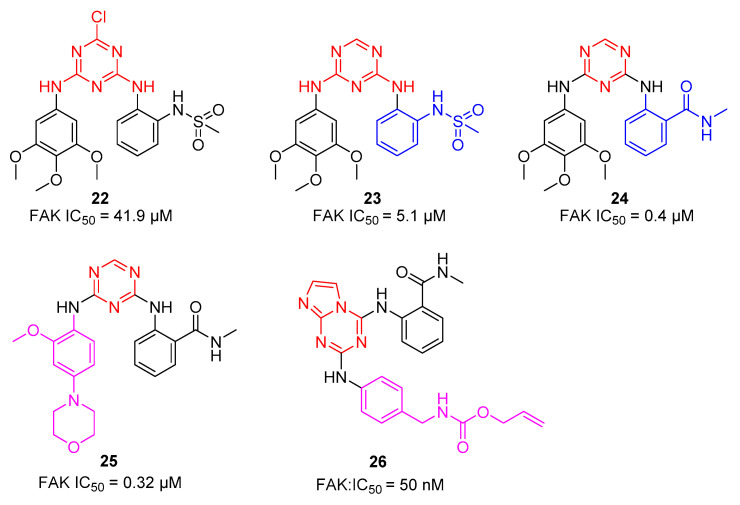
Chemical structures of FAK inhibitors.

In 2013, Chen and co-workers reported a series of diarylamino-1,3,5-triazine derivatives as FAK inhibitors [23]. As shown in Figure 5, compound **22** showed poor activity against FAK (IC_50_ = 41.9 µM) owing to the introduction of a 3,4,5-trimethoxyanilino group and a methanesulfonamide phenyl group on the 1,3,5-triazine ring. Then, they removed the chlorine atom from **22**, and compound **23** with good inhibitory activity against FAK (IC_50_ = 5.1 µM) was obtained. Moreover, they found that when the methanesulfonamide phenyl group was displaced with an amide moiety, compound **24** showed significantly increased inhibitory activity against FAK (IC_50_ = 0.4 µM). While changes in the 3,4,5-trimethoxyanilino group of compounds **24** and **25** exhibited similar inhibitory activity against FAK (IC_50_ = 0.31 µM), in order to improve the FAK kinase activity, they continued to design imidazo[1,2-a][1,3,5] triazines as novel FAK inhibitors in 2015 [24]. The new compounds displayed 10–100 nM IC_50_ values against FAK, and compound **26** (IC_50_ = 50 nM) was identified as the best inhibitor.

### 2.3. Phosphoinositide 3-Kinase Inhibition

Phosphatidylinositol 3-kinase (PI3K) is a lipid kinase, that is, a central component in the PI3K/Akt/mTOR signaling pathway [25]. The most widely studied type I PI3Ks are further subdivided into class IA (PI3Kα, β, and δ) and class IB (PI3Kγ) based on the type of catalytic structural domain. Blocking the PI3K/AKT/mTOR pathway has been widely recognized as an attractive cancer therapeutic strategy owing to its crucial role in cell growth and survival. A number of PI3K inhibitors are currently under research, including pan-PI3K-mTOR inhibitors, pan-class I PI3K inhibitors, and isoformspecific PI3K inhibitors [26]. Dimorpholino-substituted s-triazine derivatives have shown great potential in PI3K-related cancer therapy. Several promising candidates, such as ZSTK474, gedatolisib, and bimiralisib, are currently in clinical trials.

ZSTK474 is an s-triazine derivative that was selected by Zenyaku Kogyo together with more than 1500 other analogues [27]. ZSTK474 potently inhibits the four isoforms of PI3K, with IC_50_ values of 16, 44, 5, and 49 nM for PI3Kα, β, δ, and γ, respectively, suggesting that it is a pan-PI3K inhibitor [28]. Unfortunately, ZSTK-474 has been withdrawn from clinical trials due to its resistance and on-target/off-tumor side effects [29]. Therefore, the structure of ZSTK-474 needs to be optimized to improve its selective targeting of PI3K.

**Figure 6:**
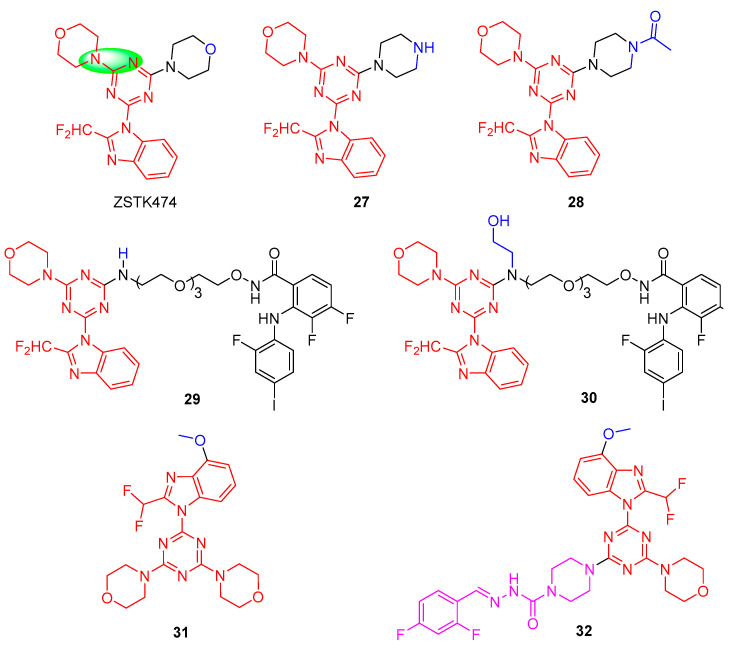
Chemical structures of PI3K inhibitors of ZSTK474 and its derivatives.

As shown in Figure 6, D. Ross et al., using ZSTK474 as a lead compound, explored compound **27** by displacing a single morpholine group in ZSTK474 with piperazine. Compound **27** showed a 36-fold reduction in its PI3Kα (IC_50_ = 180 nM) and PI3Kδ (IC_50_ = 142 nM) inhibition and a >70-fold reduction in PI3Kβ (IC_50_ = 1093 nM) and PI3Kγ (IC_50_ = 1873 nM) inhibition, indicating the sensitivity of this region towards oxygen replacement [30]. However, N-acetylation of **27** to provide **28** showed PI3K isoform inhibition (IC_50_ = 2.9−21 nM) similar to ZSTK474 (IC_50_ = 3.9−20.8 nM). To further investigate the role of the morpholine oxygen in these compounds on PI3K inhibition, they found that when the 2-aminoethyl functional groups were displaced with a single morpholine group of ZSTK474 [31], bifunctional inhibitors **29** and **30** displayed nanomolar inhibition towards PI3Kα (IC_50_ = 130 nM and 107 nM, respectively) and PI3Kδ (IC_50_ = 236 nM and 137 nM, respectively) and low micromolar inhibition for PI3Kβ and PI3Kγ (IC_50_ = 1.5−3.9 µM) in enzymatic inhibition assays. Cell viability assays demonstrated that compound **29** showed superior anti-proliferative activities over compound **30** in three tumor-derived cell lines (A375, D54, and SET-2), owing to **29** being able to inhibit downstream AKT and ERK1/2 phosphorylation.

In 2011, Shepherd and co-workers found that the introduction of a methoxy group (**31**) at the 4′-position of the 2-(difluoromethyl)-1H-benzo[d]imidazol of ZST474 could enhance interaction with the PI3Kα protein [32]. Based on this, Hou et al. designed a new 1,3,5-triazine derivative **32** containing semicarbazones as new potential PI3Kα inhibitors, **32** also further by replacing a single morpholine group of **31** with piperazine [33]. Further research indicated that compound **32** displayed excellent inhibitory activity with an IC_50_ value of 0.32 nM against PI3Kα. Meanwhile, in the U87-MG human glioblastoma xenograft model assay by intragastric administration, **32** exhibited similar antitumor activity at 20 mg/kg/day compared with ZSTK-474 at 40 mg/kg/day in vivo antitumor efficacy. However, the pharmacokinetic properties of **32** are not ideal, so the compound needs to be further modified structurally to improve its physicochemical properties.

Gedatolisib (PKI-587), another dimorpholino-substituted s-triazine derivative developed by Pfizer, is in a phase III clinical trial as a potential agent for the treatment of acute myeloid leukemia, hormone receptor-positive breast cancer, and HER2-negative breast cancer. Gedatolisib exhibits potent in an in vitro inhibitory activity against both PI3K and mTOR and displays significant antitumor effects in vivo xenograft model. However, it suffers from low selectivity over different PI3K isozymes, leading to possible off-target effects and reducing its therapeutic utility. In addition, gedatolisib must be administered by injection, which may not be suitable as a practical drug for cancer patients. Hence, structural modification of gedatolisib is desirable to develop PI3Ks and mTOR inhibitors with enhanced isozyme selectivity and metabolic stability.

**Figure 7:**
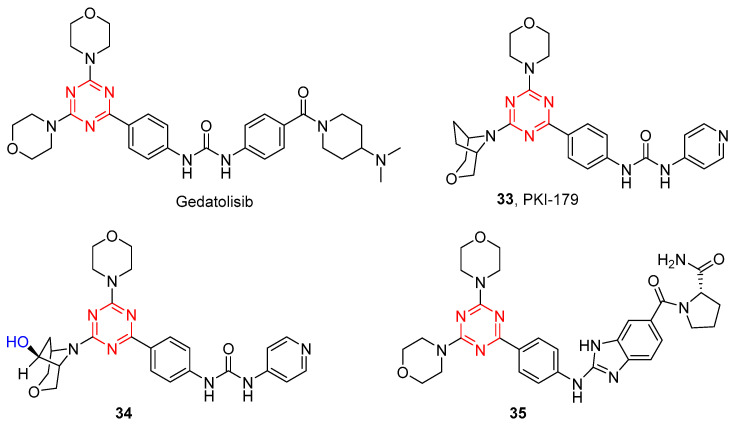
Chemical structures of PI3K inhibitors of gedatolisib and its derivatives.

The Venkatesan group has reported the bis-morpholino s-triazine-based compounds as potent dual PI3K/mTOR inhibitors [34]. Gedatolisib was found to have poor plasma levels when administered orally, which may owe to its poor permeability, low clogP (1.24), and high molecular weight (Mw: 615). In order to increase the clogP value and reduce molecular weight, Mansour et al. reported a series of mono-morpholino 1,3,5-triazine derivatives by a single morpholine group in gedatolisib that were replaced with 3-oxa-8-azabicyclo[3.2.1]octane [35]. As shown in Figure 7, **33** showed significantly increased potency against PI3Kα (IC_50_ = 8 nM) and mTOR (IC_50_ = 0.42 nM) and excellent cellular potency against both MDA-361 (IC_50_ = 22 nM) and PC3mm2 (IC_50_ = 29 nM) cell lines. Pharmacokinetic studies of **33** showed good oral bioavailability in nude mice after oral administration (10 mg/kg) and a high half-life (>60 min). Metabolite evaluation studies found that the ethylene bridge on the bridged-morpholine group was the main site of metabolism, and its metabolite structure was determined to be **34** [36]. Therefore, compound **33** is an orally effective dual PI3K/mTOR inhibitor.

Zhang et al. designed and synthesized a series of novel substituted s-triazines bearing a benzimidazole group as PI3K/mTOR inhibitors using the ring closing and scaffold hopping methods [37]. Compound **35** showed remarkable inhibitory activity against PI3Kδ with an IC_50_ value of 2.3 nM and moderate activity against other class I PI3K isoforms and mTOR (with IC_50_ values of 14.6, 34.0, 849.0, and 15.4 nM for PI3Kα, β, γ, and mTOR, respectively). Therefore, compound **35** could significantly suppress the PI3K/Akt/mTOR signaling pathway.

**Figure 8:**
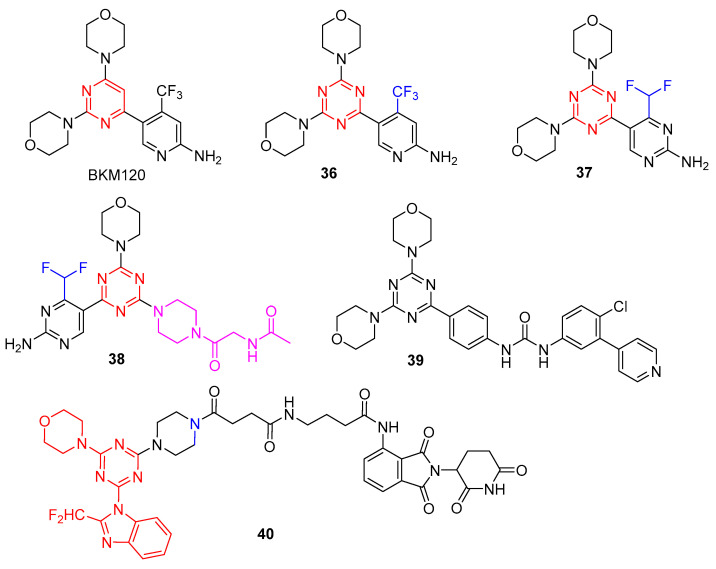
Chemical structures of PI3K inhibitors of BKM120 and its derivatives.

BKM120 is one of the clinically most advanced pan-PI3K inhibitors, but it interferes with an off-target effect on microtubule polymerization. As shown in Figure 8, Wymann et al. have identified that it differs from BKM120 by having only one atom that could be separated into discrete PI3K and tubulin activities [38]. Initially inspired by ZSTK474 and BKM120, they replaced the BKM120 core with s-triazine to yield bimiralisib (**36**), aiming to maximize compound solubility and bioavailability and avoid microtubule interactions. As expected, **36** displayed potent inhibition against pan-PI3K and showed no microtubule-destabilizing agent activity [39]. Above all, **36** is a highly selective pan-PI3K inhibitor with balanced targeting of mTOR kinase, which passed phase I studies and is now in phase II studies in relapsed and refractory lymphoma and advanced solid tumors [40,41].

Subsequently, Wymann and co-workers mainly focused on the 4-(difluoromethyl)-pyrimidin-2-amine group as an optimized residue for PI3K binding; compound **37** was obtained by substituting 4-(trifluoromethyl)-pyridine-2-amine of **36** with the group. Compound **37** showed low nanomolar affinity PI3Kα (IC_50_ = 2.2 nM) and demonstrated significant antitumor activity in a mice xenograft model at a concentration almost eight times lower than the parental phase-II inhibitor **35** [42]. Recently, starting from the lead compound **37**, they designed various potent covalent PI3Kα inhibitors by targeting the solvent-exposed cysteines at a distance > 10 Å from an ATP-site-directed core group [43]. A number of compounds bearing an acrylamide warhead and different linker modules were synthesized to assess the required warhead reactivity and the spatial trajectory of the Michael acceptor. Among these compounds, compound **38** showed the most PI3Kα enzyme inhibitory activity (IC_50_ = 1 nM) and excellent stability in rat liver microsomes.

Based on the structures of PI3K inhibitor BKM120 and Hh inhibitor vismodegib, our group reported a novel series of unsymmetrical diaryl ureas as potent inhibitors simultaneously inhibiting PI3K/Akt/mTOR and Hh signalings [44]. After biological activities were examined, compound **39** showed excellent antiproliferative activity against MDA-MB-231, T47D, and MCF-7 cells. The enzymatic activity of **39** (IC_50_ = 180 nM) against mTOR was of nanomolecular value. Furthermore, based on the structures of the PI3K inhibitor ZSTK474 and the CRBN ligand pomalidomide, we have designed a series of new small-molecule PROTACs for the degradation of PI3K [45]. Compound **40** indicated great potency on PI3Kα with an IC_50_ of 24 nM and could inhibit tumor cell proliferation by inducing autophagy instead of apoptosis or cell cycle arrest.

**Figure 9:**
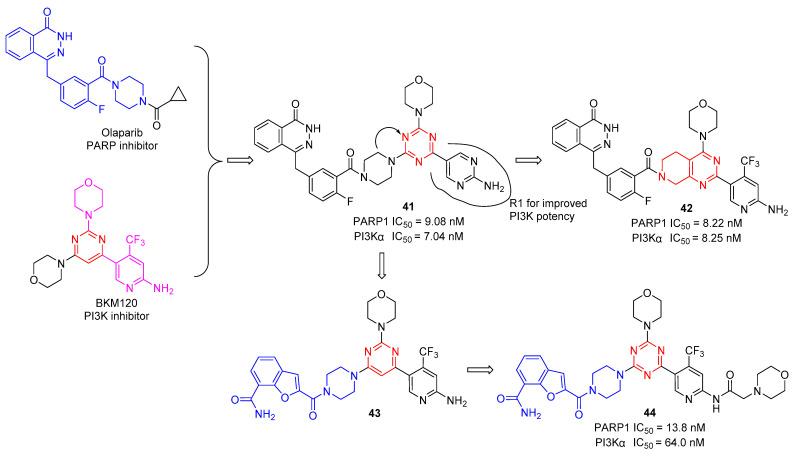
Chemical structures of s-triazine simultaneous targeting PI3K and PARP.

In 2012, Baselga and Wulf’s groups independently found that PI3K inhibition could promote HR deficiency by downregulating BRCA1/2 and could sensitize BRCA-proficient tumors to PARP inhibition, which provides an effective therapy for the combined administration of PI3K and PARP inhibitors to expand the application of PARP inhibitors [46,47]. Based on these works, Xu et al. reported a series of dual PARP/PI3K inhibitors by merging the pharmacophores of PARP and PI3K inhibitors [48]. As shown in Figure 9, structure−activity relationships (SARs) indicate that the R1 group, which serves as a hydrogen-bond donor, could be modified to change the compounds’ PI3K inhibitory activity. Several compounds were synthesized and evaluated for PARP-1 and PI3Kα inhibitory activities. As expected, all the remaining compounds showed excellent PARP-1 inhibitory activity in the low nanomolar range. The first round of structural optimization provided compound **41**, which displayed appropriate PARP-1 and PI3Kα inhibitory activities with IC_50_ values of 9.08 and 7.04 nM, respectively. Subsequently, to further optimize the 1,3,5-triazine core by a bicyclic system of tetrahydropyrido[3,4-d]pyrimidine, compound **42** displayed the most promising candidate with IC_50_ values of 8.22 and 8.25 nM against PARP-1 and PI3Kα. Next, they replaced the phthalazine moiety with a benzofuran-7-carboxamide moiety to obtain compound **43**, which exhibited good PARP-1 and PI3Ka inhibitory activity with an inhibition ratio of more than 50% at 10 nM and 100 nM, respectively. After SAR screening and experimental evaluation, the most potent compound was **44** against PARP-1 and PI3Kα with IC_50_ values of 13.8 and 64.0 nM, respectively [49].

**Figure 10:**
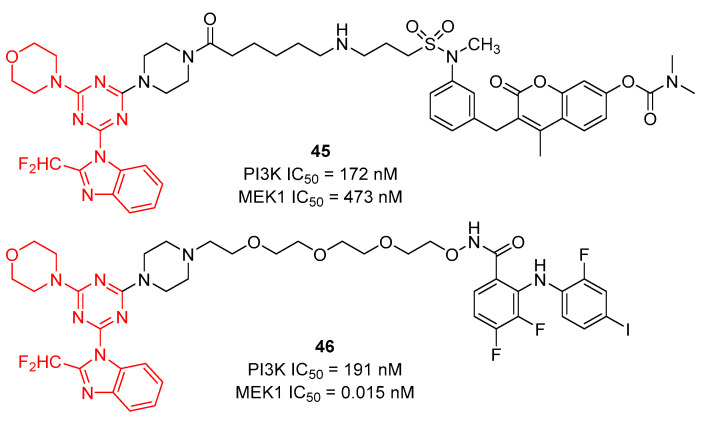
Chemical structures of PI3K and MEK bifunctional inhibitors.

The Ras/MEK/ERK and PI3K/Akt/mTOR pathways play important biological functions. MEK inhibition could promote a compensatory activation of PI3K/Akt kinase activity. As shown in Figure 10, Ross et al. reported a s-triazine analog **45** by combining the PI3K inhibitor ZSTK474 with the Raf/MEK inhibitor RO5126766. Bifunctional inhibitor **45** showed good inhibitory activity against MEK1 (IC_50_ = 473 nM) and PI3K (IC_50_ = 172 nM) [50]. However, the corresponding inhibition of pERK1/2 activity was low (35−40%) in two representative human cancer cell lines (A549, PANC-1) at the 5 μM concentration level. Recently, they continued to report a series of bifunctional MEK1/PI3K inhibitors by covalent linking the PI3K inhibitor ZSTK474 and the MEK inhibitor PD0325901 [51]. Among them, compound **46** showed excellent inhibition against MEK1 (IC_50_ = 0.015 nM) and PI3K (IC_50_ = 191 nM) and displayed a 95% and 67% inhibition of tumor ERK1/2 and Akt phosphorylation, respectively, at 2 h postadministration.

**Figure 11:**
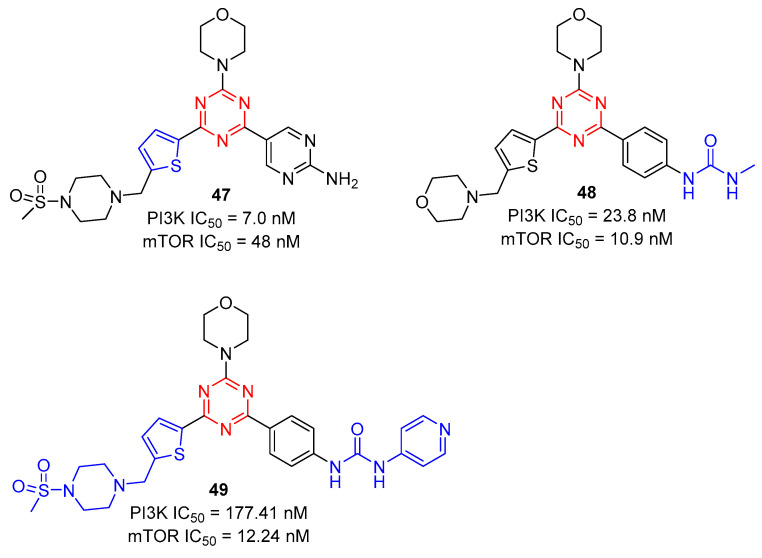
Chemical structures of PI3K and mTOR bifunctional inhibitors.

Zhu and co-workers reported 2-(thiophen-2-yl)-1,3,5-triazine derivatives **47** as PI3K and mTOR inhibitors in 2020 [52]. By experimental screening, as shown in Figure 11, compound **47** showed excellent inhibition activity of cell proliferation against A549, MCF-7, and Hela cancer cell lines with IC_50_ values of 0.20 ± 0.05 µM, 1.25 ± 0.11 µM, and 1.03 ± 0.24 µM, and potently inhibited PI3K and mTOR kinases with IC_50_ values of 7.0 and 48 nM, respectively. Further, they also reported the 2-arylurea-1,3,5-triazine derivative **48** as a PI3K and mTOR inhibitor [53]. Compound **48** exhibited potent inhibition against PI3K and mTOR kinases with IC_50_ values of 23.8 and 10.9 nM, respectively. Compound **48** was further evaluated in MCF-7 cells and MCF-7 xenograft models, which showed significant in vitro and in vivo anticancer efficacies. Besides, in order to enhance the mTOR kinase activity, they introduced aryl urea units with various substituents into the triazine core and modified it with arylurea by pyridine to obtain a series of novel thiophene-triazine derivatives bearing arylurea [54]. Among them, the inhibitory activity of **49** against PI3Kα and mTOR kinase was excellent, with IC_50_ values of 177.41 and 12.24 nM, respectively, indicating that it was a potential dual PI3Kα/mTOR inhibitor.

### 2.4. NADP^+^-Dependent Isocitrate Dehydrogenases Inhibition

**Figure 12:**
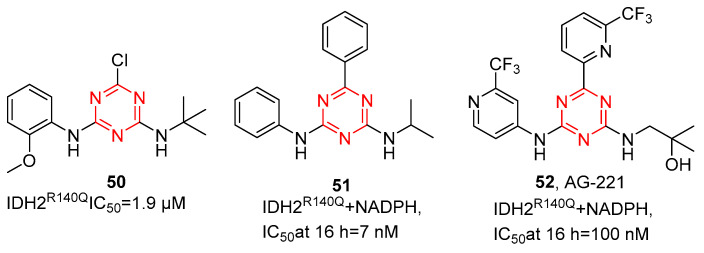
Chemical structures of IDH inhibitors of AG-221 and its derivatives.

Metabolic reprogramming is a hallmark of cancer, promoting the initiation and maintenance of tumors. The NADP^+^-dependent isocitrate dehydrogenases (IDH) are critical metabolic enzymes, converting isocitrate to α-ketoglutarate (αKG) in the tricarboxylic acid cycle. IDH mutations occur in multiple tumors, mainly in IDH1 (R132) and IDH2 (R140 and R172). In recent years, several IDH inhibitors have been approved by the FDA. Among them, AG-221 (enasidenib) is a first-in-class, orally available, small-molecule s-triazine IDH2 inhibitor [55]. Through high-throughput screening, as shown in Figure 12, Su et al. [56] reported several s-triazine compounds as effective inhibitors against IDH2^R140Q^, and the initial hit compound **50** was obtained. Compound **50** showed micromolar inhibitory potency for IDH2^R140Q^ with IC_50_ values of 1.9 µM. SAR analysis among the s-triazine analogs obtained compound **51**, the first nanomolar inhibitor of IDH2^R140Q^ (IC_50_ = 7 nM at 16 h). Although compound **51** showed potency in enzymatic and cellular assays, **51** displayed high lipophilicity, resulting in solubility-limited absorption in vivo. Additionally, it emerged poor in vitro liver microsomal stability, which translated to high clearance in vivo. Thus, they continue optimizing the substituents around the s-triazine core. Through the addition of mildly polar substituents with trifluoromethyl pyridine and 2-methyl-2-propanol to provide **52**, AG-221(**52**) showed excellent potency for 2HG inhibition, improved solubility, low clearance, and good oral bioavailability in vivo in rats, supporting the clinical trials of AG-221 in patients with IDH2 mutation.

### 2.5. Cyclin-Dependent Kinases Inhibiton

**Figure 13:**
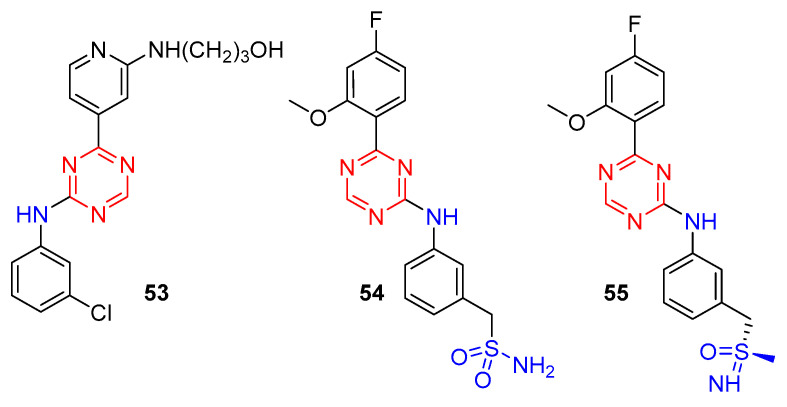
Chemical structures of s-triazine targeting CDK.

Cyclin-dependent kinases (CDKs) are members of a complex family of heterodimeric serine/threonine protein kinases that play an important role in regulating cell cycle machinery. Depending on their biological functions, CDKs can be divided into cell cycle regulatory CDKs (CDK1, CDK2, CDK4, and CDK6 are included) and transcription-associated CDKs (CDK7−9, CDK11−13, and CDK19 are included) [57]. Murray et al. have identified [1,3,5]triazine-pyridine as a new series of potent CDK inhibitors [58]. Among them, compound **53** (Figure 13) displayed excellent inhibitory potency at CDK1 (IC_50_ = 0.021 µM), CDK2 (IC_50_ = 0.007 µM), and CDK5 (IC_50_ = 0.003 µM) and submicromolar potency at CDK4 (IC_50_ = 0.308 µM), CDK6 (IC_50_ = 0.356 µM), and CDK7 (IC_50_ = 0.126 µM). The broad spectrum of potent CDK inhibitory activities and the in vitro and in vivo antitumor efficacy of **53** may render it as a valuable pharmacological tool in elucidating the complex roles of CDK signaling pathways.

Positive transcription elongation factor b (PTEFb) is a hetero-dimer of CDK9 and one of four cyclin partners: cyclin T1, cyclin K, cyclin T2a, or cyclin T2b. Deregulated kinase activity of CDK9 of the PTEFb hetero-dimer is associated with cancer. Starting from lead compound **54**, Lucking et al. have optimized structurally characterized by an unuaual benzyl sulfoximine group to obtain **55** [59]. Through kinase selectivity, physicochemical properties, DMPK properties, in vitro and in vivo efficacy, **55** is the first potent and highly selective PTEFb/CDK9 (IC_50_ CDK9/CycT1: 13 nm, ratio of IC_50_ values CDK2/CDK9: 100) inhibitor to enter clinical trials for the treatment of cancer.

### 2.6. Others

**Figure 14:**
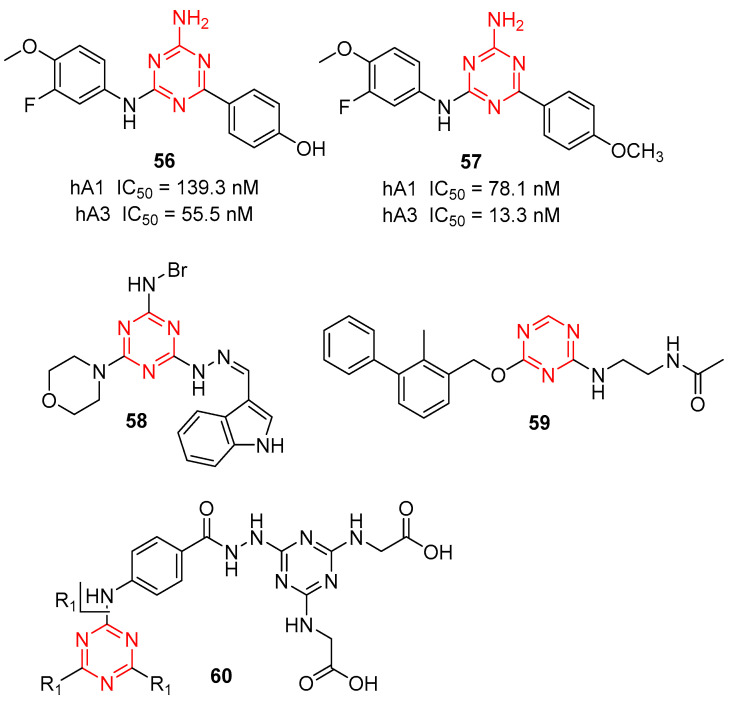
Chemical structures of hAR inhibitors **56**, **57**, Hsp90 inhibitors **58**, PD-L1. Inhibitors **59**, and MMP and VEGF inhibitors **60**.

Human adenosine receptors (hARs) can be classified into four subtypes: hA1, hA2A, hA2B, and hA3. All four belong to the G protein-coupled receptor (GPCR) family, and each has a different pharmacological profile, tissue distribution, and function. Langmead et al., in an effort to find a novel hit by virtual screening, reported several compounds containing 1,3,5-triazine that bind to hA1 and hA2A. Subsequently, 1,3,5-triazine analogs were identified as potent human adenosine receptor antagonists for Parkinson’s disease [60,61]. To modify s-triazine to produce selective ligands for subtypes other than hA2A. Yu et al. designed and synthesized novel 1,3,5-triazine derivatives targeting the hA1 and hA3 adenosine receptors for cancer therapy [62]. As shown in Figure 14, compounds **56** and **57** showed good binding affinity to both hA1 (IC_50_ = 139.3 nM, 78.1 nM, respectively) and hA3 AR (IC_50_ = 55.5 nM, 13.3 nM, respectively) and could inhibit cell viability, leading to cell death in lung cancer cell lines.

Heat shock protein 90 (Hsp90), a kind of molecular chaperone, is widely expressed and highly conserved in cells. DCZ5248 (**58**, Figure 14), a triazine derivative by the Lou group, is a novel Hsp90 inhibitor that directly binds to the Hsp90 protein and does not induce a heat shock response [63], and identified **58** was a dual inhibitor of both Hsp90 and late-autophagy with potent antitumor activity against colon cancer cells in vitro and in vivo.

Programmed cell death protein 1 (PD-1) and its ligand PD-L1 comprise immune checkpoints located on T-cells. The PD-1/PD-L1 axis is hijacked by tumor cells to suppress immune surveillance. At present, inhibition of the PD-1/PD-L1 axis by monoclonal antibodies has achieved remarkable success and is approved for cancer. However, a number of small molecules targeting PD-L1 have been reported [64]. Marinelli and co-workers have identified 2,4,6-tri- and 2,4-disubstituted 1,3,5-triazines as PD-L1 inhibitors [65]. Among them, compound **59** demonstrated to strongly bind with the PD-L1 protein with a nanomolar IC_50_ of 0.115 µM and not to PD-1, as shown in Figure 14.

Matrix metalloproteinases (MMPs) are a characteristic family of zinc-dependent endopeptidases that could promote extracellular matrix turnover, tumor growth, angiogenesis, and metastasis. Considering MMP-9 could promote cell migration and trigger the angiogenic switch during carcinogenesis via expression of vascular endothelial growth factor (VEGF), therefore, Khattab et al. developed a series of star-shaped triazine-based dendrimers as MMP and VEGF inhibitors [66]. As shown in Figure 14, the most active anticancer dendrimer **60** showed potent MMP-9, -10, and -13 inhibitors with IC_50_ values of 156, 145, and 124 nM, respectively. Furthermore, **60** suppressed the correlated oncogenic mediators, VEGF expression, induced apoptosis (>75%), and inhibited tumor cell migration (∼84%) in Caco-2 cells.

## 3. Conclusions and Prospect

A growing body of evidence supports the fact that s-triazines are universal analogues and could exert anticancer effects through diverse signal pathways. Consequently, discovering s-triazine inhibitors has become a very active and fast-paced area of research over the past several decades. There has been remarkable progress in the s-triazine structurally modified field as a result of collective advances made in assay development, high-throughput screening, structural biology, medicinal chemistry, and cellular and animal models. Since 1990, when the first triazine-containing anticancer drugs were approved, numerous triazine-containing anticancer drugs have entered clinical trials. For instance, PI3K/mTOR inhibitors [gedatolisib (approved by FDA), ZSTK-474 (II), and bimiralisib (II)] and IDH2 inhibitors [enasidenib (approved by FDA)] are all in human clinical trials, underscoring the rapid progress made in the field.

The SARs of s-triazine illustrated that (1) modifications at the C-4 and C-6 positions of the 1,3,5-triazin-2(1H)-one moiety could enhance the Topo activity. (2) The 1,3,5-triazine scaffold modified by more hydrophobic side chains would additionally occupy the H3 pocket of BTK, which could improve inhibitory activities and selectivity for BTK. (3) EGFR inhibitors usually consist of three parts: (a) 1,3,5-triazine containing a nitrogen substitute moiety of the hinge binder, which mainly forms hydrogen bonds with the ATP binding pocket; (b) phenyl part of the hydrophobic group, which usually extends to the back pocket area of the kinase and could improve its selectivity; (c) solubilized long chain, which could modulate the biophysical and antiproliferation activity. (4) 1,3,5-triazine FAK inhibitors have similar structure containing a 2-aminobenzene group and orthoformamide or sulfonamide; extending chain length could increase FAK activities. (5) Dimorpholino-substituted s-triazine is necessary for most PI3K and mTOR inhibitors due to the fact that the oxygen atom of morpholine in the hinge binder could form a key hydrogen bond with the NH of Val 851 and Val 2240. (6) Mildly polar substituents around the s-triazine core could improve IDH inhibition.

While there has been significant progress, there is still much to be achieved in the s-triazine derivatives field. As above, s-triazines could target the topoisomerases, tyrosine kinases, phosphoinositide 3-kinases, NADP^+^-dependent isocitrate dehydrogenases, and cyclin-dependent kinases. However, only PI3K and IDH inhibitors have entered clinical trials. Compared to the large number of papers about the antitumor activities of s-triazine derivatives, the reports of s-triazine derivatives targeting other proteins are limited, and further study is needed. Although s-triazines displayed good activity against both solid tumors and blood tumors, most of the s-triazine drugs mainly displayed potent activity against leukemia (enasidenib) and lymphocarcinoma (altretamine) diseases. Therefore, identification and optimization of efficient s-triazine derivatives to expand the indications for solid tumors are still needed. The study of the mode of s-triazines has yielded exciting results; while the mechanism of action of drugs is complex for cancer therapy, an abundant understanding of the drugs’ mechanisms is advantageous to the s-triazines’ structure optimization.

In this review, we thoroughly covered the discovery, structure optimization, and application of s-triazines for investigating the physiological functions and disease implications of the target proteins. We highlighted key advances and opportunities in the s-triazines field. We hope that this review will be a useful resource and inspire new and original discoveries.

## Data Availability

No new data were created or analyzed in this review. Data sharing is not applicable to this manuscript.
